# Methodological quality of systematic reviews referenced in clinical practice guidelines for the treatment of opioid use disorder

**DOI:** 10.1371/journal.pone.0181927

**Published:** 2017-08-03

**Authors:** Andrew Ross, Justin Rankin, Jason Beaman, Kelly Murray, Philip Sinnett, Ross Riddle, Jordan Haskins, Matt Vassar

**Affiliations:** Oklahoma State University Center for Health Sciences, Tulsa, Oklahoma, United States of America; University of Illinois-Chicago, UNITED STATES

## Abstract

**Introduction:**

With efforts to combat opioid use disorder, there is an increased interest in clinical practice guidelines (CPGs) for opioid use disorder treatments. No literature exists examining the quality of systematic reviews used in opioid use disorder CPGs. This study aims to describe the methodological quality and reporting clarity of systematic reviews (SRs) used to create CPGs for opioid use disorder.

**Methods:**

From June to July 2016 guideline clearinghouses and medical literature databases were searched for relevant CPGs used in the treatment of opioid use disorder. Included CPGs must have been recognized by a national organization. SRs from the reference section of each CPG was scored by using AMSTAR (a measurement tool to assess the methodological quality of systematic reviews) tool and PRISMA (preferred reporting items for systematic reviews and meta-analyses) checklist.

**Results:**

Seventeen CPGs from 2006–2016 were included in the review. From these, 57 unique SRs were extracted. SRS comprised 0.28% to 17.92% of all references found in the CPGs. All SRs obtained moderate or high methodological quality score on the AMSTAR tool. All reviews met at least 70% of PRISMA criteria. In PRISMA, underperforming areas included accurate title labeling, protocol registration, and risk of bias. Underperforming areas in AMSTAR included conflicts of interest, funding, and publication bias. A positive correlation was found between AMSTAR and PRISMA scores (r = .79).

**Conclusion:**

Although the SRs in the CPGs were of good quality, there are still areas for improvement. Systematic reviewers should consult PRISMA and AMSTAR when conducting and reporting reviews. It is important for CPG developers to consider methodological quality as a factor when developing CPG recommendations, recognizing that the quality of systematic reviews underpinning guidelines does not necessarily correspond to the quality of the guideline itself.

## Introduction

Clinical practice guidelines (CPGs) help practitioners manage patients in an effective and systematic way, and they assist in making evidence-based decisions related to diagnosis and treatment. The role of CPGs as key decision support tools is highlighted by their near-universal integration into daily clinical practice [1]. By providing evidence and standards for optimal care, CPGs have been shown to improve outcomes, reduce health care costs, and lower the use of health care services in chronic conditions [2]. However, CPGs display a range of differences in their development processes, reporting quality, methodological quality, and content [3,4] and they often suffer from bias introduced by both direct and indirect conflicts of interest [5]. Consequently, the universal acceptance of the recommendations found in CPGs is problematic unless a critical and unbiased evaluation of supporting evidence has been performed. Owing to the ineffective and inefficient use of limited resources and the potential harm to patients imposed by the adoption of guidelines with questionable validity and quality, it becomes increasingly important for clinicians to scrutinize the methodological quality, strength, and direction of the studies used to support individual recommendations [4,6,7].

Although the grading of evidence in CPGs has improved over time, only a minority of CPGs explicitly score the strength of the evidence on which they are based [8]. This situation highlights the need for clinicians to scrutinize the clarity and methodological quality of the evidence for specific recommendations as well as the quality of the guideline as a whole [4].

Among CPGs, guidelines on opioid prescribing and the treatment of opioid use disorder are controversial. Perspectives on opioid guideline direction have evolved over decades, and they vacillate between endorsement and restriction of opioid treatment, with controversy being driven by concerns about safety, effectiveness, and risk of use [5,9]. The continuing controversy over the treatment of opioid use disorder is further complicated by the economic burden of opioid dependence, which is estimated to be in excess of US$55 billion in 2007 [8,10]. Driven by excessive medical and prescription expenses, health care costs composed nearly half of this burden [10]. The societal burden has only worsened in more recent years, with deaths due to opioid overdose having increased 14% from 2013 to 2014 and having doubled between 2000 and 2014 [11]. Spurred by a rapidly increasing economic burden and facing an American population that consumes 80% of the world’s opioid supply [12], lawmakers in 49 states have instituted prescription drug monitoring programs (PDMPs) in an effort to minimize use and the diversion of controlled substances [13]. However, the effect of these programs themselves remains largely controversial, with conflicting reports on the impact of PDMPs on opioid prescription, overdose, and overdose mortality [14–16]. After continued failure of non-treatment programs for opioid use disorder, a $1.1 billion budget was recently approved by the U.S. Congress to expand access to medical treatment of opioid use disorders [17]. However, although new guidelines on prescribing opioids have been recently released [9], no literature examining the quality of current CPGs exists for opioid use disorder or for systematic reviews (SRs) that serve as underlying evidence for these CPGs. In addition, much controversy surrounds the manufacturers of opioid medications [18], and an ever-growing body of evidence links financial relationships and conflicts of interest in guideline development (e.g., by reducing the study quality or quality of the underlying evidence) [18–23]. As such, researchers must be able to evaluate the quality of evidence underlying CPGs because any translation of industry bias into patient care could prove detrimental.

This study aims to (1) identify the methodological quality and clarity of reporting in SRs underlying CPGs for opioid use disorder, (2) describe the variation in SR quality in CPGs published by different professional medical associations, and (3) outline the variation in SR quality of opioid use disorder CPGs between the United States and other countries with opioid use disorder treatment guidelines.

## Methods

### Protocol development and registration

Search strategies, eligibility criteria, and data abstraction were pre-specified in the research protocol developed and piloted *a priori*. This study did not meet the regulatory definition of human subject research as defined in 45 CFR 46.102(d) and (f) of the Department of Health and Human Services’ Code of Federal Regulations (“45 CFR 46”, 2016), and it was not subject to Institutional Review Board oversight. To ensure best practices in data abstraction and management, we consulted Li et al 2015 [24], the Cochrane Handbook for Systematic Reviews of Interventions [25], and the National Academies of Science, Engineering and Medicine’s (previously the Institute of Medicine) Standards for Systematic Reviews [26]. Preferred Reporting Items for Systematic Reviews and Meta-Analyses (PRISMA) guidelines [27] for systematic review and Statistical Analyses and Methods in the Published Literature (SAMPL) guidelines [28] for descriptive statistics were applied when relevant. Before initiating the study, we registered it with the University hospital Medical Information Network Clinical Trial Registry (UMIN-CTR, UMIN000023126), and study data are publically available on figshare (https://dx.doi.org/10.6084/m9.figshare.3496781).

### Identification of eligible clinical practice guidelines

One author (A.R.) searched the National Guideline Clearinghouse, the Scottish Intercollegiate Guideline Network (SIGN), the Australian Clinical Practice Guidelines, Guidelines International Network (GIN), Google Scholar, Google News Alerts, and Google for relevant CPGs using keywords including opioid dependence and opioid use disorder. A second author (M.V.) performed a PubMed search using this search string: ((((((("opioid-related disorders"[MeSH Major Topic] OR "buprenorphine"[MeSH Terms]) OR buprenorphine[Title/Abstract]) OR "methadone"[MeSH Terms]) OR methadone[Title/Abstract]) OR opioid[Title/Abstract]) OR opiate[Title/Abstract]) AND ((abuse[Title/Abstract] OR addiction[Title/Abstract]) OR dependence[Title/Abstract])) AND ("guidelines as topic"[MeSH Terms:noexp] OR "practice guidelines as topic"[MeSH Terms] OR "health planning guidelines"[MeSH Terms] OR guideline[pt] OR practice guideline[pt]). This search string was based on a Cochrane systematic review search strategy designed to identify studies for opioid dependence [29]. We modified the Canadian Agency for Drugs and Technologies in Health’s Information Services Filters Working Group’s search hedge for locating clinical practice guidelines in PubMed [30].

After identifying relevant CPGs from these searches, A.R. reviewed their reference sections to identify additional CPGs that were not previously located. We included CPGs published between January 1, 2006, and June 1, 2016. We defined the term “clinical practice guideline” *a priori* as “statements that include recommendations intended to optimize patient care that are informed by a systematic review of evidence and an assessment of the benefits and harms of alternative care options,” using the National Academies of Science, Engineering and Medicine’s definition [31]. To be eligible, CPGs had to have been recognized by a national, governmental, or professional organization. For CPGs with multiple versions, we used the most recent version. If CPGs published addendums, the addendum was also included. To reduce errors through translation, we opted *a priori* to only include guidelines published in English; however, no guidelines were ultimately excluded based on this criterion.

### Identification of eligible systematic reviews

Two authors (A.R. and J.R.) searched the reference section of each guideline with keyword searches (e.g., “systematic”, “meta-”, “rev”, “Cochrane”) to identify SRs or meta-analyses. No disagreements occurred between the authors, so no third party adjudication was needed. *A priori*, we used the National Academies of Science, Engineering and Medicine’s definition for an SR: *“*a scientific investigation that focuses on a specific question and uses explicit, pre-specified scientific methods to identify, select, assess, and summarize the findings of similar but separate studies. It may include a quantitative synthesis (meta-analysis), depending on the available data" [31]. This definition was selected to be as inclusive as possible. We did not use definitions that set standards for the number of databases searched or required a meta-analysis to avoid conflict with our assessment tools. To be eligible, a SR had to have been referenced in an eligible guideline.

### Data abstraction and scoring

Prior to abstraction and scoring, all authors were trained using video modules and detailed tutorials developed by P.S. that outlined the process. Authors then completed a piloted practice exercise to become acquainted with scoring and abstraction. Four authors (A.R., J.R., R.R., and J.H.) independently completed abstraction and scoring on a subset of SRs, using piloted abstraction forms. Following scoring, each score was verified by a second author. Disagreements were resolved by consensus between the pair. A third-party adjudication process was established in the protocol but not needed. This scoring and verification process was followed throughout. For each SR, authors abstracted the following study characteristics: year of publication, participant population, intervention, number of primary studies, sample size of primary studies, and research design of primary studies. Authors then independently scored each SR using the PRISMA checklist and the AMSTAR tool, described in the following sections.

### AMSTAR Tool

AMSTAR (A Measurement Tool to Assess Systematic Reviews) is an 11-item measure used to determine the quality of SRs [32]. AMSTAR has been acknowledged as a valid and reliable tool with high interrater reliability, construct validity, and feasibility [33]. We used AMSTAR, instead of R-AMSTAR (the revised version), because AMSTAR is more easily applied. R-AMSTAR has also been criticized for inherent subjectivity and repetitiveness [34]. We applied recommended revisions made by Burda et al 2016 [35] to AMSTAR. These changes focus on improving validity, reliability, and usability in assessing methodological quality, and they include changes in order of items, wording of items and instructions, and modifications to the focus of original items 7, 8, and 11. These recommendations also address aspects noted to be problematic in numerous studies and improve specificity to methodological quality over quality of reporting or risk of bias [35,36]. However, the additional item described by Burda et al. 2016 [35] was not included since subgroup analyses are not applicable to all SRs and meta-analyses. The addition of the item complicates scoring of the tool. Additional instructions were provided to reviewers if modified instructions were unclear. Each item was initially answered with a “criteria met,” “criteria not met,” “criteria partially met,” and “not applicable.” The answer “not applicable” was only available on item 10 (concerning small study effects), and it was selected if the SR included fewer than 10 primary studies. This modification was made since funnel plot methods lack power to detect true asymmetry when the number of primary studies is fewer than 10 [37]. Points were then awarded for each answer as follows: 1 point for criteria met and 0 points for other answers. The total score was then categorized into three categories based on their score: Low (0–3), Moderate (4–7), High (8–11) [38].

### PRISMA checklist

We assessed the clarity of reporting in eligible SRs using the PRISMA (Preferred Reporting Items for SRs and Meta-Analysis) checklist [39]. It has been acknowledged for its usefulness in critically appraising SRs and meta-analyses even though it was originally developed for authors to improve the quality of their reviews [40]. However, the quality of reporting does not necessarily equate to methodological quality in SRs, necessitating use of tools that independently assess both qualities [39,41]. The assessment contains 27 items designed to evaluate reporting quality. Each checklist item was answered with “criteria met,” “criteria partially met,” or “criteria not met” based on the completeness of reporting. Unlike AMSTAR we allowed partial credit on PRISMA items since completeness of reporting was thought to be more adequately accounted for using this method. For example, Item 7 of the PRISMA checklist states, “Did the systematic review describe all information in the search and date last searched?”. In this case, a systematic review was assigned a partial score of one if it described all information sources in the search but did not state the date last searched. Points were then awarded as follows: 2 points for criteria met, 1 point for criteria partially met, and 0 points for criteria not met.

## Results

### Search results

**Fig 1:**
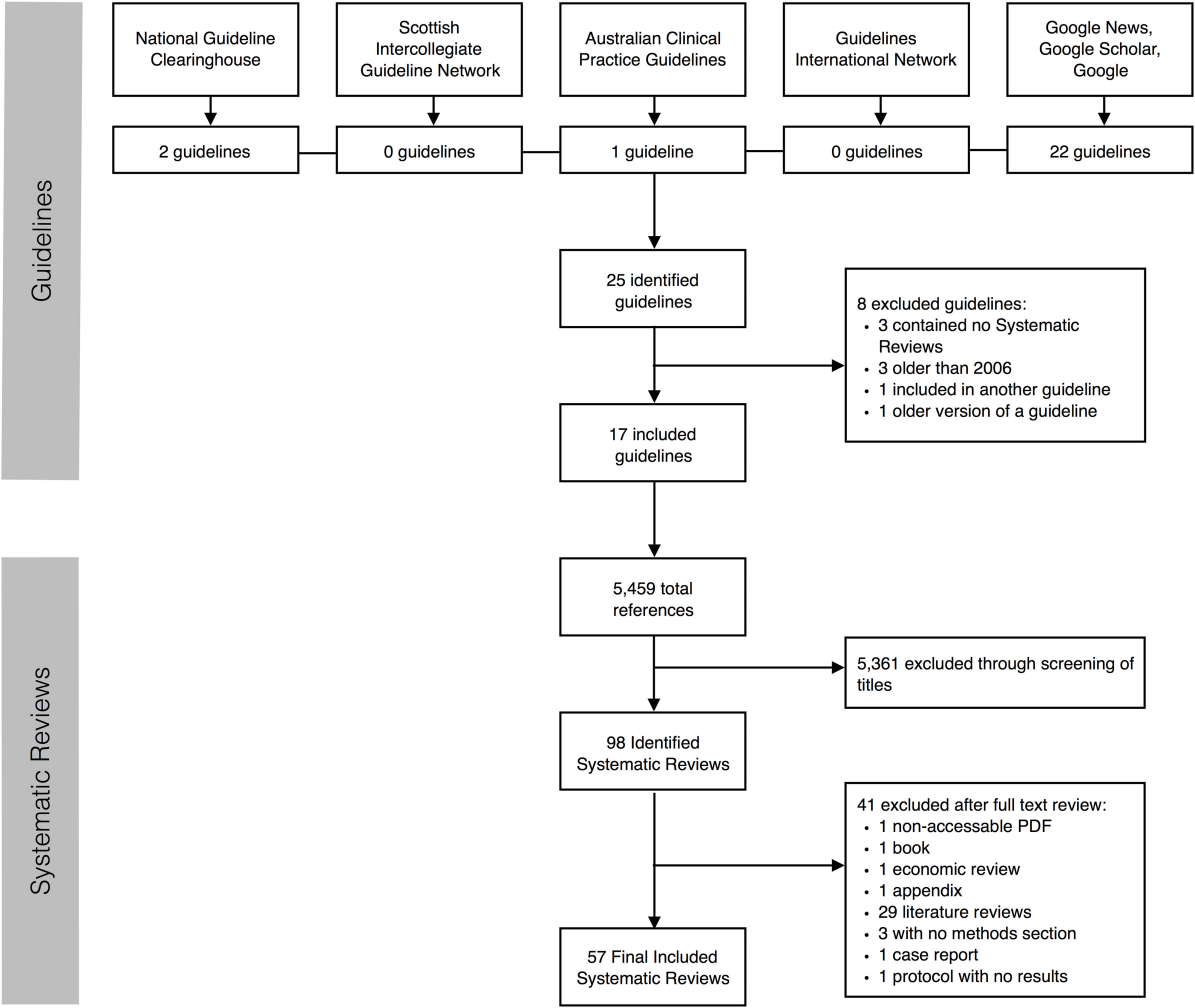
PRISMA flow diagram of excluded studies.

Our guideline search initially yielded 25 guidelines (Fig 1). Eight were excluded for the following reasons: three did not reference any SRs, three were published prior to 2006, one was already included in another guideline, and one was an outdated version of a guideline already included. A total of 17 guidelines were included in this study. From these 17 guidelines, there were 5,459 references. After screening the references using the titles, and in some cases, the abstract, 5,361 references were excluded. Of the 98 studies that proceeded to full-text review, 41 studies were excluded. Reasons for exclusion are listed in Fig 1. Ultimately, 57 unique SRs were included in this study of which 22 were included in more than one guideline (Table 1). Characteristics of included guidelines are presented in Table 2.

**Table 1:** Systematic reviews used across guidelines.

Reference	PRISMA(Mean)	AMSTAR(Total Score)	ANG	WFSBP	VG	WHO	BAP	ASAM	APA	RCGP	CAMH	MMT	ANGB	SMG	MPG	VaDoD	PCOT	NZG	NICE
Mattick 2014	0.88	9(11)	**X**	**X**	**X**	**X**	**X**		**X**	**X**	**X**	**X**	**X**				**X**		
Amato 2011a	0.94	10(11)	**X**	**X**	**X**	**X**	**X**		**X**						**X**	**X**			
Gowing 2009a	0.91	10(11)	**X**	**X**	**X**	**x**	**X**			**X**	**X**		**X**						
Mattick 2009	0.86	9(11)	**X**	**X**	**X**	**X**	**X**	**X**	**X**			**X**							
Minozzi 2011	0.89	10(11)	**X**		**X**	**X**	**X**	**X**								**X**			
Faggiano 2002	0.88	9(11)	**X**	**X**	**X**		**X**							**X**					
Ferri 2011	0.90	9(10)	**X**	**X**	**X**	**X**	**X**												
Amato 2013	0.94	10(11)	**X**		**X**	**X**						**X**							
Amato 2011b	0.93	9(11)	**X**	**X**	**X**	**X**													
Gowing 2010	0.83	9(10)	**X**	**X**		**X**		**X**											
Gowing 2014	0.94	11(11)		**X**	**X**	**X**				**X**									
Cleary 2010	1.00	9(11)	**X**	**X**				**X**											
Connock 2007	0.98	9(11)		**X**							**X**								**X**
Day 2005	0.80	8(10)	**X**	**X**		**X**													
Dutra 2008	0.80	5(11)		**X**				**X**							**X**				
Barnett 2001	0.66	4(10)		**X**						**X**									
Castells 2009	0.92	10(11)		**X**	**X**														
Gowing 2009b	0.93	9(10)	**X**			**X**													
Gowing 2011	0.88	9(11)	**X**		**X**														
Prendergast 2000	0.54	4(11)		**X**					**X**										
Prochaska 2004	0.84	9(11)	**X**					**X**											
West 1999	0.62	4(10)		**X**									**X**						
Adi 2007	0.91	9(11)		**X**															
Arias 2006	0.54	3(11)		**X**															
Bao 2009	0.64	4(11)												**X**					
Bargagli 2007	0.90	8(11)				**X**													
Chou 2014	0.78	7(11)			**X**														
Chou 2013	0.87	9(10)	**X**																
Hunt 2013	0.96	11(11)		**X**															
Drake 2008	0.58	5(11)		**X**															
Dunn 2001	0.74	6(11)	**X**																
Kellias 2009	0.76	6(11)	**X**																
Farre 2002	0.72	8(11)							**X**										
Ferri 2013	0.85	8(10)			**X**														
Goldner 2014	0.94	8(11)			**X**														
Hedrich 2012	0.82	7(11)	**X**																
Horspool 2008	0.52	7(10)									**X**								
Lobmaier 2008	0.94	10(11)		**X**															
MacArthur 2012	0.96	9(11)			**X**														
Malta 2008	0.76	7(11)	**X**																
Marsch 1998	0.63	4(11)								**X**									
Merrall 2010	0.72	5(10)			**X**														
Minozzi 2014a	0.94	5(10)					**X**												
Minozzi 2014b	0.90	10(10)			**X**														
Minozzi 2013	0.91	9(10)					**X**												
Nunes 2004	0.89	9(11)	**X**																
Osborn 2010a	0.96	9(10)	**X**																
Osborn 2010b	0.94	9(10)	**X**																
Pani 2013	0.69	7(10)																**X**	
Pani 2010	0.87	9(10)	**X**																
Prendergast 2001	0.72	7(11)		**X**															
Roozen 2004	0.94	8(11)			**X**														
Rosner 2010a	0.98	11(11)		**X**															
Rosner 2008	0.82	6(11)		**X**															
Rosner 2010b	0.84	11(11)	**X**																
Srivastava 2008	0.66	4(11)										**X**							
Strand 2013	0.64	4(11)	**X**																
**PRISMA Mean Score**			0.8	0.8	0.9	0.9	0.9	0.8	0.8	0.8	0.8	0.8	0.8	0.7	0.9	0.9	0.9	0.7	1
**AMSTAR Total Score**			8.8	8.1	9.5	9.5	9.6	8.6	8	7.7	8..8	8	7.8	6.5	7.5	10	9	7	9

**Abbreviations for Guidelines**: PCOT-Guideline for Physicians Working in California Opioid Treatment Programs; CAMH- Center for Addiction and Mental Health Buprenorphine/Naloxone for Opioid Dependence; WHO- World Health Organization Guidelines for the Psychosocially Assisted Pharmacological Treatment of Opioid Dependence; SMG- State of Michigan- Medication Assisted Treatment Guidelines for Opioid Use Disorder; ANG-Australian National Guidelines for Medication-Assisted Treatment of Opioid Dependence; VG- Vancouver A guideline for the Clinical Management of Opioid Addiction; ASAM- American Society of Addiction Medicine National Practice Guidelines for the use of medications in the treatment of addiction involving opioid use; BAP- British Association for Psychopharmacology updated guidelines: evidence-based guideline for the pharmacological management of substance abuse, harmful use, addiction and comorbidity: recommendations from BAP; MMT- Methadone Maintenance Treatment Program Standards and Clinical Guidelines; NZG- New Zealand Practice Guidelines for Opioid Substitution Treatment; APA- American Psychiatry Association Practice Guideline for the Treatment of Patients with Substance use Disorders; RCGP- Royal College of General Practitioners Guidance for the use of substitute prescribing in the treatment of opioid dependence in primary care; WFSBP- The World Federations of Societies of Biological Psychiatry Guidelines for the Biological Treatment Substance Use and Related Disorder. Part 2: Opioid Dependence; ANGB- Australia National clinical guidelines and procedures for the use of buprenorphine in the maintenance treatment of opioid dependence; MPG- Magellan Clinical Practice Guidelines for the assessment and treatment of Patients with substance use disorder; Va/DoD- Department of Veteran Affairs/Department of Defense; Management of SUG-2015; NICE- National Institute for Health and Care Excellence Methadone and buprenorphine for the management of opioid dependence. Citations for all referenced systematic reviews are on figshare(https://dx.doi.org/10.6084/m9.figshare.3496781).

**Table 2:** Guideline characteristics.

Abbreviation	Guidelines	Year of publication	Geographical area of impact	Number of references in each guideline	Number of Systematic Reviews	Percentage of Systematic Review
PCOT	Guideline for Physicians Working in California Opioid Treatment Programs	2008	United States of America	78	1	1.28%
CAMH	Centre for Addiction and Mental Health-Buprenorphine/Naloxone for Opioid Dependence	2011	Canada	359	4	1.11%
WHO	World Health Organization- Guidelines for the Psychosocially Assisted Pharmacological Treatment of Opioid Dependence	2009	Other (Switzerland)	327	13	3.98%
SMG	State of Michigan- Medication Assisted Treatment Guidelines for Opioid Use disorder	2014	United States of America	88	2	2.27%
ANG	Australian- National Guidelines for Medication-Assisted Treatment of Opioid Dependence	2014	Australia	318	26	8.18%
VG	Vancouver- A guideline for the Clinical Management of Opioid Addiction	2015	Canada	106	19	17.92%
ASAM	American Society of Addiction Medicine- National Practice Guidelines for the use of medications in the treament of addiction involving opioid use	2015	United States of America	173	6	3.47%
BAP	British Association for Psychopharmacology updated guidelines: evidence-based guidelines for the pharmacological management of substance abuse, harmful use, addiction and comorbidity: recommendations from BAP	2012	United Kingdom	583	9	1.54%
MMT	Methadone Maintenance Treatment Program Standards and Clinical Guidelines	2011	Canada	227	4	1.76%
NZG	New Zealand Practice Guidelines for Opioid Substitution Treatment	2014	New Zealand	64	1	1.56%
APA	American Psychiatric Association- Practice Guideline for the Treatment of Patients With Substance Use Disorders	2006	United States of America	1789	5	0.28%
RCGP	Royal College of General Practitioners- Guidance for the use of substitute prescribing in the treatment of opioid dependence in primary care	2011	United Kingdom	118	5	4.24%
WFSBP	World Federation of Societies of Biological Psychiatry- Guidelines for the Biological Treatment of Substance Use and Related Disorders. Part 2: Opioid Dependence	2011	Other	331	25	7.55%
ANGB	Australia- National clinical guidelines and procedures for the use of buprenorphine in the maintenance treatment of opioid dependence	2006	Australia	90	3	3.33%
MPG	Magellan- Clinical Practice Guidelines for the assessment and treatment of patients with substance use disorders	2015	United States of America	228	2	0.88%
VaDoD	Department of Veteran Affairs/ Department of Defense- Management of SUD- 2015	2015	United States of America	327	2	0.61%
NICE	National Institute for Health and Care Excellence- Methadone and buprenorphine for the management of opioid dependence	2007	Other	254	1	0.39%

### Scoring results

Scores for both PRISMA and AMSTAR were averaged for all SRs in each guideline. For the PRISMA checklist, each item was averaged to give a percentage of items met (Table 3). For the AMSTAR tool, each number was added for each guideline for a total score out of 11, and a quality rating was assigned (Table 4). For both tools, the average number of SRs addressing each item across guidelines was calculated to identify problematic items. The following results are based solely on the SRs referenced in the CPGs and are not evaluations of the guidelines themselves.

**Table 3:** Summary of PRISMA scores by guidelines.

PRISMA Item	PCOT	CAMH	WHO	SMG	ANG	VG	ASAM	BAP	MMT	NZG	APA	RCGP	WFSBP	ANGB	MPG	VaDoD	NICE	Mean
1.Systematic review, meta-analysis, or both in the title?	0.00	0.50	0.23	0.50	0.42	0.32	0.50	0.00	0.50	0.00	0.40	0.40	0.56	0.33	0.50	0.00	1.00	**0.36**
2.Structured summary in the abstract?	1.00	0.75	1.00	1.00	1.00	1.00	1.00	1.00	1.00	1.00	0.80	1.00	0.92	1.00	1.00	1.00	1.00	**0.97**
3.Rationale for review in the introduction?	1.00	1.00	1.00	1.00	1.00	1.00	1.00	1.00	1.00	1.00	1.00	1.00	1.00	1.00	1.00	1.00	1.00	**1.00**
4.Objectives statement in the introduction?	1.00	1.00	1.00	1.00	1.00	1.00	1.00	1.00	1.00	1.00	1.00	1.00	1.00	1.00	1.00	1.00	1.00	**1.00**
5.Protocol registration information provided?	0.50	0.50	0.38	0.25	0.46	0.37	0.33	0.44	0.25	0.00	0.20	0.30	0.34	0.33	0.25	0.50	1.00	**0.38**
6.Methods for eligibility criteria included?	1.00	1.00	1.00	1.00	1.00	1.00	1.00	1.00	1.00	1.00	1.00	1.00	1.00	1.00	1.00	1.00	1.00	**1.00**
7.Information sources in the methods?	1.00	0.88	0.88	0.75	0.90	0.89	0.92	1.00	0.75	1.00	0.90	0.90	0.86	1.00	1.00	1.00	1.00	**0.92**
8.Full search strategy provided?	1.00	1.00	1.00	0.75	0.85	0.95	0.92	1.00	0.88	1.00	0.60	0.60	0.80	1.00	1.00	1.00	1.00	**0.90**
9.Process of study selection provided?	1.00	0.75	0.96	0.50	1.00	0.95	1.00	1.00	1.00	1.00	0.80	0.80	0.82	0.67	1.00	1.00	1.00	**0.90**
10.Process of data extraction provided?	1.00	0.75	1.00	0.50	0.90	0.92	1.00	1.00	1.00	1.00	1.00	0.70	0.84	0.67	1.00	1.00	1.00	**0.90**
11.List and define all variables for which data were sought?	1.00	0.75	1.00	1.00	0.92	1.00	1.00	1.00	1.00	1.00	1.00	1.00	0.98	0.83	1.00	1.00	1.00	**0.97**
12.Methods for risk of bias in individual studies provided?	1.00	1.00	1.00	0.50	0.77	0.95	0.67	1.00	0.75	1.00	0.60	0.60	0.68	0.67	0.50	1.00	1.00	**0.80**
13.Methods for principal study measures provided?	1.00	0.75	0.92	1.00	0.88	0.95	1.00	1.00	0.88	1.00	1.00	0.90	0.92	1.00	1.00	1.00	1.00	**0.95**
14.Methods for synthesis of results provided?	1.00	0.75	0.88	1.00	0.77	0.89	0.83	1.00	0.88	1.00	0.80	1.00	0.80	1.00	0.50	1.00	0.50	**0.86**
15.Methods for risk of bias across studies provided (publication bias)?	0.00	0.75	0.69	0.25	0.56	0.76	0.83	0.72	0.50	1.00	0.40	0.60	0.66	0.33	1.00	1.00	1.00	**0.65**
16.Methods of additional analyses provided?	N/A	0.50	0.62	0.00	0.50	0.53	0.50	1.00	0.25	1.00	1.00	1.00	0.48	1.00	0.50	1.00	1.00	**0.68**
17.Description of studies included/excluded?	1.00	0.88	0.88	1.00	0.92	0.97	0.75	0.94	1.00	1.00	0.80	0.60	0.82	0.67	0.50	1.00	1.00	**0.87**
18.Study characteristics for the included studies provided?	1.00	1.00	1.00	1.00	1.00	1.00	1.00	1.00	1.00	0.00	0.90	0.90	0.90	1.00	1.00	1.00	1.00	**0.92**
19.Risk of bias in individual studies assessed?	1.00	0.75	0.96	0.50	0.75	0.95	0.58	1.00	0.75	0.00	0.60	0.60	0.66	0.67	0.50	1.00	1.00	**0.72**
20.Results of the individual studies presented ideally in a forest plot?	1.00	0.75	1.00	1.00	0.98	0.95	1.00	1.00	1.00	0.00	0.80	1.00	0.94	1.00	1.00	1.00	1.00	**0.91**
21.Clear synthesis of the results with proper measurements in consistency?	1.00	0.75	1.00	1.00	1.00	0.95	1.00	1.00	1.00	0.00	0.90	0.80	0.94	1.00	1.00	1.00	1.00	**0.90**
22.Risk of bias across individual studies assessed (publication bias)?	0.00	0.50	0.69	0.00	0.56	0.68	0.75	0.61	0.38	0.00	0.40	0.40	0.60	0.33	1.00	0.75	1.00	**0.51**
23.Results of any additional analyses provided?	N/A	0.50	0.54	0.00	0.46	0.42	0.33	0.80	0.25	0.00	1.00	1.00	0.48	1.00	1.00	0.50	1.00	**0.58**
24.Summary of evidence in the discussion?	1.00	1.00	1.00	0.75	1.00	1.00	1.00	1.00	1.00	1.00	1.00	1.00	0.96	1.00	1.00	1.00	1.00	**0.98**
25.Discussion of limitations of the study?	1.00	0.88	0.88	1.00	0.90	0.92	0.83	0.83	0.75	0.50	0.80	0.70	0.90	0.83	1.00	1.00	1.00	**0.87**
26.Discussion of the implications and future research?	1.00	1.00	1.00	1.00	1.00	1.00	1.00	1.00	0.88	1.00	1.00	1.00	0.96	0.83	1.00	1.00	1.00	**0.98**
27.Funding source and roles of the authors provided?	1.00	0.75	1.00	0.75	0.98	1.00	1.00	1.00	0.75	1.00	1.00	1.00	0.92	0.67	1.00	1.00	1.00	**0.93**
**Mean**	**0.86**	**0.79**	**0.87**	**0.70**	**0.83**	**0.86**	**0.84**	**0.90**	**0.79**	**0.69**	**0.80**	**0.81**	**0.81**	**0.81**	**0.86**	**0.92**	**0.98**	

**Abbreviations**: PCOT-Guideline for Physicians Working in California Opioid Treatment Programs; CAMH- Centre for Addiction and Mental Health Buprenorphine/Naloxone for Opioid Dependence; WHO- World Health Organization Guidelines for the Psychosocially Assisted Pharmacological Treatment of Opioid Dependence; SMG- State of Michigan- Medication Assisted Treatment Guidelines for Opioid Use Disorder; ANG-Australian National Guidelines for Medication-Assisted Treatment of Opioid Dependence; VG- Vancouver A guideline for the Clinical Management of Opioid Addiction; ASAM- American Society of Addiction Medicine National Practice Guidelines for the use of medications in the treatment of addiction involving opioid use; BAP- British Association for Psychopharmacology updated guidelines: evidence-based guideline for the pharmacological management of substance abuse, harmful use, addiction and comorbidity: recommendations from BAP; MMT- Methadone Maintenance Treatment Program Standards and Clinical Guidelines; NZG- New Zealand Practice Guidelines for Opioid Substitution Treatment; APA- American Psychiatric Association Practice Guideline for the Treatment of Patients with Substance use Disorders; RCGP- Royal College of General Practitioners Guidance for the use of substitute prescribing in the treatment of opioid dependence in primary care; WFSBP- The World Federations of Societies of Biological Psychiatry Guidelines for the Biological Treatment Substance Use and Related Disorder. Part 2: Opioid Dependence; ANGB- Australia National clinical guidelines and procedures for the use of buprenorphine in the maintenance treatment of opioid dependence; MPG- Magellan Clinical Practice Guidelines for the assessment and treatment of Patients with substance use disorder; Va/DoD- Department of Veteran Affairs/Department of Defense Management of SUG-2015; NICE- National Institute for Health and Care Excellence Methadone and buprenorphine for the management of opioid dependence.

**Table 4:** Summary of AMSTAR scores by guideline.

AMSTAR Item	PCOT	CAMH	WHO	SMG	ANG	VG	ASAM	BAP	MMT	NZG	APA	RCGP	WFSBP	ANGB	MPG	VaDoD	NICE	Mean
1. Was an 'a priori" design provided?	1.0	1.0	0.9	1.0	0.9	0.9	1.0	1.0	1.0	1.0	1.0	1.0	1.0	1.0	1.0	1.0	1.0	**0.99**
2. Was a comprehensive literature search performed?	1.0	1.0	1.0	1.0	1.0	0.9	1.0	1.0	1.0	1.0	0.8	0.6	0.8	1.0	1.0	1.0	1.0	**0.95**
3. Was relevant grey literature included in the review?	1.0	1.0	1.0	0.5	0.9	0.8	1.0	1.0	0.8	1.0	1.0	0.6	0.9	0.7	1.0	1.0	1.0	**0.89**
4. Was there duplicate study selection and data extraction?	1.0	0.8	0.9	0.5	0.8	0.9	0.8	1.0	0.8	1.0	0.8	0.6	0.7	0.7	0.5	1.0	1.0	**0.81**
5. Was a list of studies (included and excluded) provided?	1.0	0.8	0.9	0.5	0.7	0.7	0.5	1.0	0.8	1.0	0.8	0.6	0.6	0.7	0.5	1.0	1.0	**0.77**
6. Were the characteristics of the included studies provided?	1.0	1.0	1.0	1.0	1.0	1.0	1.0	1.0	1.0	0.0	1.0	1.0	1.0	1.0	1.0	1.0	1.0	**0.94**
7. Was the scientific quality of the included studies used appropriately in formulating the conclusions?	1.0	1.0	1.0	0.5	0.8	0.9	0.8	1.0	0.8	1.0	0.6	0.6	0.7	0.7	0.5	1.0	1.0	**0.82**
8. Was the scientific quality of the included studies used appropriately in formulating the conclusions?	1.0	1.0	0.9	0.5	0.8	1.0	0.8	1.0	0.8	0.0	0.8	1.0	0.7	0.7	0.5	1.0	1.0	**0.80**
9. Were the methods used to combine the findings of the study appropriate?	1.0	1.0	1.0	1.0	1.0	0.9	0.8	1.0	1.0	1.0	1.0	1.0	0.9	1.0	0.5	1.0	1.0	**0.95**
10. Was the likelihood of publication bias assessed?	0.0	0.3	0.8	0.0	0.7	0.7	0.8	0.5	0.3	N/A	0.2	0.5	0.6	0.5	1.0	1.0	0.0	**0.49**
11. Were potential conflicts of interest included?	0.0	0.0	0.1	0.0	0.2	0.2	0.0	0.1	0.0	0.0	0.0	0.2	0.2	0.0	0.0	0.0	0.0	**0.05**
**Total Score**	**9.0**	**8.8**	**9.5**	**6.5**	**8.8**	**9.1**	**8.6**	**9.6**	**8.0**	**7.0**	**8.0**	**7.7**	**8.1**	**7.8**	**7.5**	**10.0**	**9.0**	
**Quality Rating**	**High**	**High**	**High**	**Moderate**	**High**	**High**	**High**	**High**	**High**	**Moderate**	**High**	**Moderate**	**High**	**Moderate**	**Moderate**	**High**	**High**	

**Abbreviations**: PCOT-Guideline for Physicians Working in California Opioid Treatment Programs; CAMH- Center for Addiction and Mental Health Buprenorphine/Naloxone for Opioid Dependence; WHO- World Health Organization Guidelines for the Psychosocially Assisted Pharmacological Treatment of Opioid Dependence; SMG- State of Michigan- Medication Assisted Treatment Guidelines for Opioid Use Disorder; ANG-Australian National Guidelines for Medication-Assisted Treatment of Opioid Dependence; VG- Vancouver A guideline for the Clinical Management of Opioid Addiction; ASAM- American Society of Addiction Medicine National Practice Guidelines for the use of medications in the treatment of addiction involving opioid use; BAP- British Association for Psychopharmacology updated guidelines: evidence-based guideline for the pharmacological management of substance abuse, harmful use, addiction and comorbidity: recommendations from BAP; MMT- Methadone Maintenance Treatment Program Standards and Clinical Guidelines; NZG- New Zealand Practice Guidelines for Opioid Substitution Treatment; APA- American Psychiatric Association Practice Guideline for the Treatment of Patients with Substance use Disorders; RCGP- Royal College of General Practitioners Guidance for the use of substitute prescribing in the treatment of opioid dependence in primary care; WFSBP- The World Federations of Societies of Biological Psychiatry Guidelines for the Biological Treatment Substance Use and Related Disorder. Part 2: Opioid Dependence; ANGB- Australia National clinical guidelines and procedures for the use of buprenorphine in the maintenance treatment of opioid dependence; MPG- Magellan Clinical Practice Guidelines for the assessment and treatment of Patients with substance use disorder; Va/DoD- Department of Veteran Affairs/Department of Defense Management of SUG-2015; NICE- National Institute for Health and Care Excellence Methadone and buprenorphine for the management of opioid dependence. Cut points for quality rating: 0-3(Low), 4-7(Moderate), 8-11(High).

No guideline received a low SR methodological quality rating from the AMSTAR tool for their SRs (Table 4) and none scored below a 70% based on the PRISMA criteria (Table 3). Five guidelines received a moderate quality rating on the AMSTAR tool. Of the five guidelines, MPG [42] had the highest adherence to PRISMA criteria (86%) and NZG [43] had the lowest (69%). Twelve guidelines received a high methodological quality rating on the AMSTAR tool. Of the 12 guidelines, NICE [44] and VaDoD [45] had the highest adherence to PRISMA criteria (98% and 92%, respectively). Both guidelines had a low number of SRs in their references ($\bar{x}$ = 1, 0.39%; $\bar{x}$ = 2, 0.61%; respectively). MMT [46] and CAMH [47] had the lowest adherence to PRISMA criteria (79%) in this category; however, they contained more SRs in their references ($\bar{x}$ = 4, 1.76%;$\bar{x}$ = 4, 1.11%; respectively) than NICE [44] and VaDoD [45].

Across all guidelines, items 1 (systematic review or meta-analysis in title, $\bar{x}$ = 0.36) and 5 (protocol registration, $\bar{x}$ = 0.38) of the PRISMA criteria showed a need for improvement. PCOT [48], NZG [43], BAP [49], and VaDoD [45] had an average of zero, meaning that none of their SRs addressed this item, and NICE [44] had an average of one for item 1 (systematic review or meta-analysis in the title). NZG [43] had an average of zero for item 5 (protocol registration) and NICE [44] had an average of one. However, NICE [44], NZG [43], and PCOT [48] only contained one SR. Items 3, 4, and 6 (rational for review, objective statement, eligibility criteria; respectively) of the PRISMA criteria showed remarkable adherence (100%) by each guideline (Table 2). For the AMSTAR tool, items 10 (publication bias,$\bar{x}$ = 0.50) and 11 (conflict of interest,$\bar{x}$ = 0.11) showed the lowest adherence across all guidelines. NICE [44], SMG [50], and PCOT [48] averaged zero for both items. MPG [42] fully adhered to item 10 (publication bias,$\bar{x}$ = 1) but did not adhere to item 11 (conflict of interest,$\bar{x}$ = 0). Items 1 and 2(a priori design, comprehensive literature search; respectively) of the AMSTAR tool had the highest adherence across all guidelines ($\bar{x}$ = 0.99 and $\bar{x}$ = 0.95, respectively). Despite this high adherence, RCGP [51] showed a lower adherence ($\bar{x}$ = 0.6) to item 2(a priori design) (Table 3).

**Fig 2:**
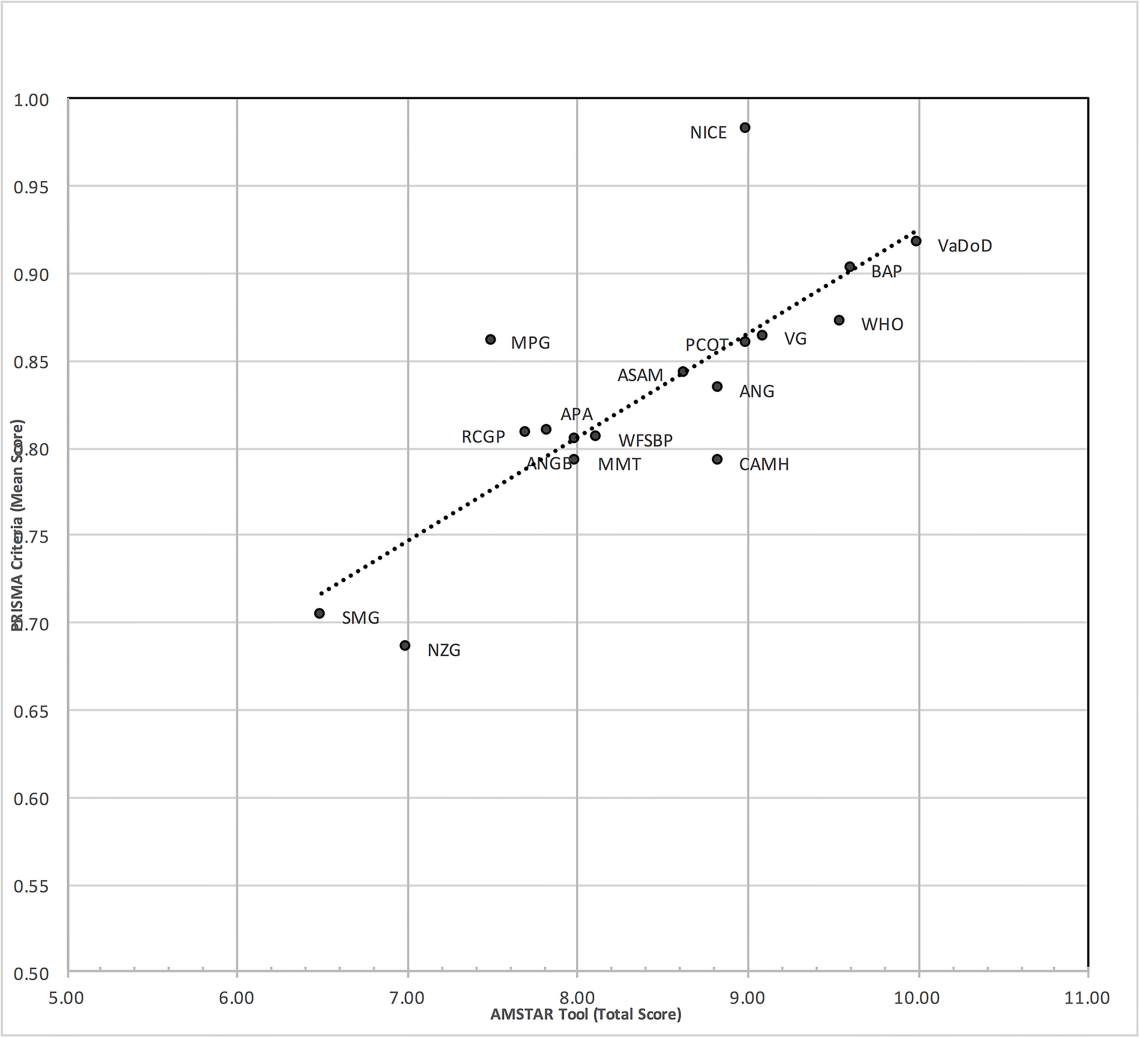
Scatterplot of AMSTAR and PRISMA scores.

Overall, the PRISMA and AMSTAR scores were highly correlated (r = .79) (Fig 2). For example, VaDoD [45], had an average of 0.9 on the PRISMA criteria and a total score of 10/11 on the AMSTAR tool. Likewise, SMG [50] had the lowest score on the PRISMA criteria (= 0.7) and was associated with a low score on the AMSTAR tool (6.5/11, 59%).

**Fig 3:**
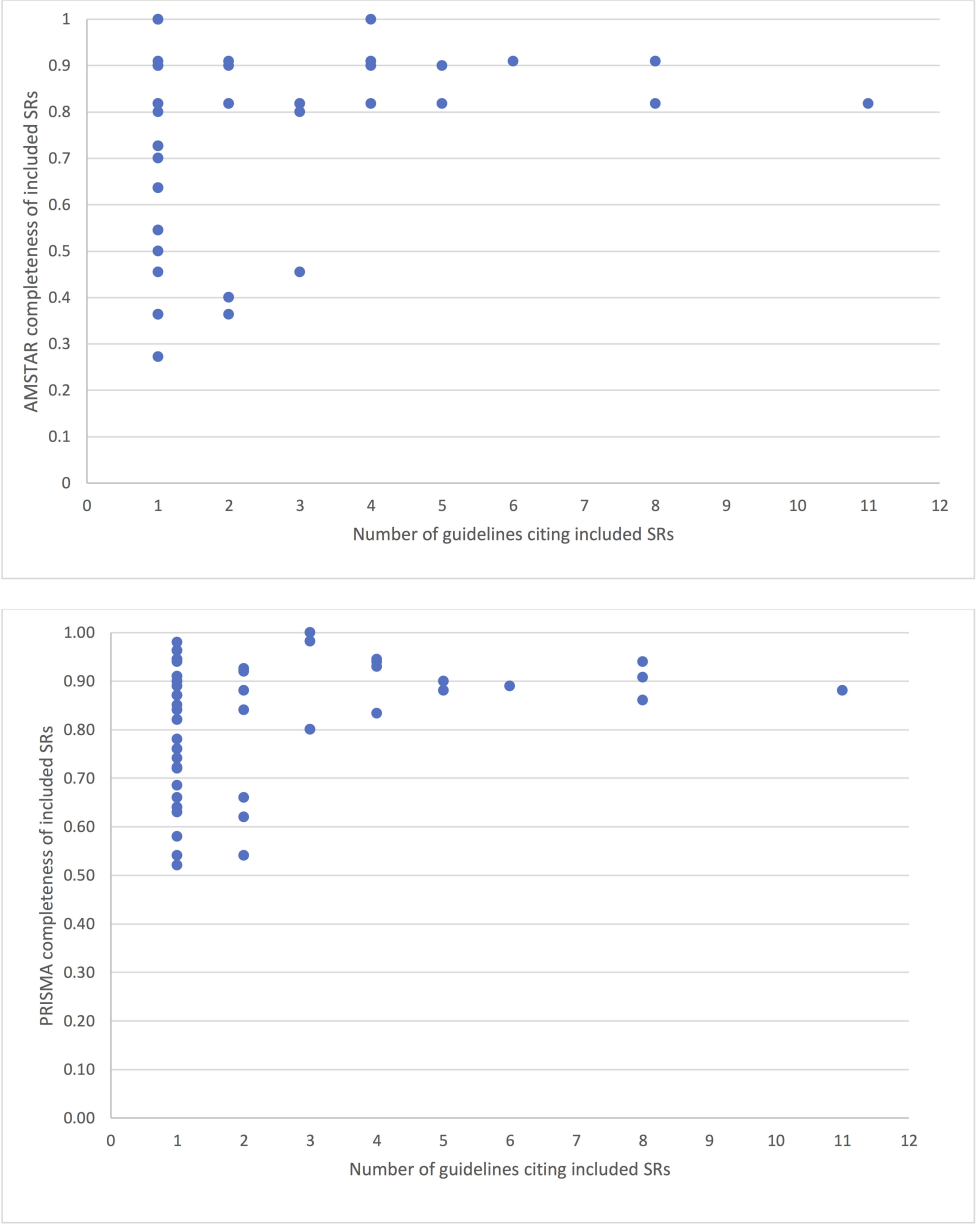
PRISMA and AMSTAR scores and use of SRs across guidelines.

The five most referenced SRs were Mattick et al. 2014 [29] (11/17,65%), Amato et al. 2011a [52], Gowing et al. [53](8/17,47%), Mattick et al. 2009 [54](8/17,47%), and Minozzi et al. 2011 [55] (6/17,35%). Of these, Amato et al. 2011a [52] had the highest adherence to PRISMA criteria (94%). This was followed by Gowing et al. 2009a [53](91%), Minozzi et al 2011 [55](89%), Mattick et al. 2014 [29](88%), Mattick et al. 2009 [54](86%). Minozzi et al. 2011 [55]. Gowing et al. 2009a [53] and Amato et al. 2011a [52] had a total score of 10/11 on the AMSTAR tool. Mattick et al. 2009 [54] and Mattick et al. 2014 [29] had a total score of 9/11 on the AMSTAR tool. Thirty-five SRs were only referenced in one guideline. The most referenced SR, Mattick et al. 2014 [29], was not referenced in the following guidelines: ASAM [56], SMG [50], MPG [42], NZG [43], NICE [44] and VaDoD [45]. Of these guidelines, ASAM [56], MPG [42], and VaDoD [45] could have used this reference because they were published in 2015 (Table 1). Fig 3 depicts the AMSTAR and PRISMA scores by the number of times the systematic reviews were included in guidelines. In general, systematic reviews referenced in multiple guidelines had consistently higher AMSTAR and PRISMA scores. Greater variability was found in systematic reviews cited in only 1 or 2 guidelines.

## Discussion

Our study found that the overall methodological and reporting quality of SRs included in guidelines for treatment of opioid use disorder was moderate to high. There are, however, still areas in which SRs could be improved. Results from our study suggest that disclosed funding of studies, assessment of publication bias, and reporting a registered protocol were the most problematic areas. Each of them plays a known role in SR outcomes.

Funding of the primary studies was underreported; however, evidence suggests that funding plays a role in the magnitude of clinical effect sizes. One recent study of funding in cardiovascular trials found that half of the industry-funded studies reported positive outcomes compared with only one-fifth of the non–industry-funded studies [57]. Possible explanations for favorable results in industry-sponsored research include selective funding of superior drugs, poor quality research, not selecting an appropriate comparator, and publication bias [58]. Unknown funding sources of primary studies within an SR can thus affect the summary effect size of the SR if funding source, rather than the intervention, contributes to bias in the outcomes of these trials.

Publication bias is a second contributor to inflated summary effects of SRs. Reliance on statistically significant outcomes from the published literature likely exaggerates the treatment effects across studies since nonsignificant findings are smaller in magnitude, less often published, and less often included in SRs. A substantial body of evidence has demonstrated the negative effects of publication bias on clinical outcomes [59–63]. Conducting a publication bias evaluation is not always recommended. In some cases, too few studies are available to conduct these evaluations. For funnel plot–based tests, at least 10 primary studies are needed for sufficient power to detect true asymmetry [25,37]. Furthermore, in cases of reviews of diagnostic test accuracy, diagnostic odds ratios typically diverge substantially from 1, and funnel plot methods are not recommended. In cases like these, systematic reviewers should acknowledge the potential for publication bias and provide a rationale for omitting the assessments.

A final area of weakness among SRs was the lack of a pre-established protocol or registration. These mechanisms serve to limit arbitrary decision making by reviewers, to allow for investigation of selective reporting bias (between registry/protocol and the published review), to foster collaborations, and to reduce research waste [64]. There are currently limited options for registering SRs. Perhaps the most widely used registry is PROSPERO, developed by the Centre for Reviews and Dissemination and funded by the National Institute for Health Research. PROSPERO catalogues prospectively registered SRs of health-related outcomes in the fields of health and social care, welfare, public health, education, crime, justice, and international development. Features of the registration are maintained as a permanent record to limit selective reporting bias [65]. Some clinical trial registries also permit prospective SR registration. For example, Japan’s University hospital Medical Information Network (UMIN) trial registry permits these registrations. SR protocols are also being published in academic journals as another measure of accountability and transparency. The journals *Systematic Reviews* and *BMJ Open* frequently publishes these protocols. In the event that the review is registered in PROSPERO, the protocol only receives editorial review by the handling editor of *Systematic Reviews* [66]. The PRISMA-P checklist was recently published as a means to guide reviewers on the completeness of reporting information for SR protocols. Hopefully, these mechanisms will promote greater use of prospective registration and protocol development.

We found that systematic reviews cited more frequently in guidelines had higher PRISMA and AMSTAR scores, suggesting greater rigor when conducting and reporting these systematic reviews. This finding is important, given that clinicians use these guidelines to inform patient care. These findings also have implications for future research, as there is limited evidence on the quality of systematic reviews referenced in guidelines and whether those referenced more frequently are of higher (or lower) methodological quality than systematic reviews referenced by fewer guidelines or not referenced at all.

There have recently been calls for more extensive partnerships between SR teams and guideline development bodies to align activities [31,67]. These partnerships would facilitate improved use of SRs in developing guideline recommendations because guideline developers would be more aware of the existence of SRs. In turn, systematic reviewers would have a greater sense of the clinical questions to address in their reviews. It has also been suggested that systematic reviewers should participate on guideline development teams to enhance application of research findings and bridge the gap between research and practice [67]. PROSPERO and GIN are currently working together to foster these important collaborations.

Our study contained limitations. One limitation was that only SRs included in CPGs were scored. If a SR was not clearly identified by title as a SR or meta-analysis, it may have been missed during the screening process. The reviews were also identified in the reference section of each CPG and were not tied to specific recommendations, since many CPGs failed to associate their practice recommendations with particular references. Furthermore, although SRs are important in development of CPGs, there are many other types of research that contribute to CPG development. Evaluating the quality of the SRs is not an indication of the quality of the CPG as a whole. Although the AMSTAR tool is a validated tool to assess methodological quality, our use of modified AMSTAR items based on recent recommendations has yet to be empirically validated [35]. The recommended changes theoretically improve specificity to methodological quality and address known issues present in the original AMSTAR tool, but the degree to which this tool, when modified, measures methodological quality is as yet unknown. Furthermore, we assumed equal weighting of items for both AMSTAR and PRISMA, and it is likely that particular items making up these measures have more relevance to guideline development panels than others. Last, as some systematic reviews were referenced in multiple guidelines, there is the possibility for bias in guideline scores.

Closer examination of CPGs for opioid addiction is timely and important given recent movement toward solutions for opioid addiction. The Comprehensive Addiction and Recovery Act of 2016 became legislation on July 22, 2016. This law was enacted to address improper use of prescription opioids and illicit opioid substances, like heroin, and address better access to treatment and recovery options [68]. This act will likely affect clinical treatment recommendations, thus a review of the underlying SR evidence in CPGs for opioid use disorder will provide greater confidence in current recommendations. The need may lead this area of medicine, and the need has never been greater for appropriate production of CPGs based on sound evidence from adequately structured SRs.

## References

[1] Bodenheimer T, Wagner EH, Grumbach K. Improving primary care for patients with chronic illness. JAMA. 2002 10 9;288(14):1775–9. 12365965 10.1001/jama.288.14.1775

[2] Bodenheimer T, Wagner EH, Grumbach K. Improving primary care for patients with chronic illness: the chronic care model, Part 2. JAMA. 2002 10 16;288(15):1909–14. 12377092 10.1001/jama.288.15.1909

[3] AGREE Collaboration. Development and validation of an international appraisal instrument for assessing the quality of clinical practice guidelines: the AGREE project. Qual Saf Health Care. 2003 2;12(1):18–23. doi: 10.1136/qhc.12.1.18 12571340 10.1136/qhc.12.1.18 PMC1743672

[4] Graham ID, Harrison MB. Evaluation and adaptation of clinical practice guidelines. Evid Based Nurs. 2005 7;8(3):68–72. 16021701 10.1136/ebn.8.3.68

[5] Boyd EA, Akl EA, Baumann M, Curtis JR, Field MJ, Jaeschke R, et al. Guideline Funding and Conflicts of Interest: Article 4 in Integrating and Coordinating Efforts in COPD Guideline Development. An Official ATS/ERS Workshop Report. Proc Am Thorac Soc. 2012 Dec 15;9(5):234–42. 10.1513/pats.201208-057ST 23256165

[6] Andrews JC, Schünemann HJ, Oxman AD, Pottie K, Meerpohl JJ, Coello PA, et al GRADE guidelines: 15. Going from evidence to recommendation—determinants of a recommendation’s direction and strength. J Clin Epidemiol. 2013;66(7):726–35. doi: 10.1016/j.jclinepi.2013.02.003 23570745 10.1016/j.jclinepi.2013.02.003

[7] Andrews J, Guyatt G, Oxman AD, Alderson P, Dahm P, Falck-Ytter Y, et al GRADE guidelines: 14. Going from evidence to recommendations: the significance and presentation of recommendations. J Clin Epidemiol. 2013 7;66(7):719–25. doi: 10.1016/j.jclinepi.2012.03.013 23312392 10.1016/j.jclinepi.2012.03.013

[8] Grilli R, Magrini N, Penna A, Mura G, Liberati A. Practice guidelines developed by specialty societies: the need for a critical appraisal. Lancet. 2000 1 8;355(9198):103–6. doi: 10.1016/S0140-6736(99)02171-6 10675167 10.1016/S0140-6736(99)02171-6

[9] Dowell D, Haegerich TM, Chou R. CDC Guideline for Prescribing Opioids for Chronic Pain—United States, 2016. MMWR Recomm Rep. 2016 3 18;65(1):1–49. doi: 10.15585/mmwr.rr6501e1 26987082 10.15585/mmwr.rr6501e1

[10] Birnbaum HG, White AG, Schiller M, Waldman T, Cleveland JM, Roland CL. Societal costs of prescription opioid abuse, dependence, and misuse in the United States. Pain Med. 2011 4;12(4):657–67. doi: 10.1111/j.1526-4637.2011.01075.x 21392250 10.1111/j.1526-4637.2011.01075.x

[11] Rudd RA, Aleshire N, Zibbell JE, Matthew Gladden R. Increases in Drug and Opioid Overdose Deaths—United States, 2000–2014. Am J Transplant. 2016 4 1;16(4):1323–7. 10.15585/mmwr.mm6450a3 26720857

[12] Manchikanti L, Singh A. Therapeutic opioids: a ten-year perspective on the complexities and complications of the escalating use, abuse, and nonmedical use of opioids. Pain Physician. 2008 3;11(2 Suppl):S63–88. 18443641

[13] Meyer R, Patel AM, Rattana SK, Quock TP, Mody SH. Prescription opioid abuse: a literature review of the clinical and economic burden in the United States. Popul Health Manag. 2014 12;17(6):372–87. doi: 10.1089/pop.2013.0098 25075734 10.1089/pop.2013.0098 PMC4273187

[14] Green TC, Zaller N, Rich J, Bowman S, Friedmann P. Revisiting Paulozzi et al.’s “Prescription Drug Monitoring Programs and Death Rates from Drug Overdose”: Table 1. Pain Med. 2011 6 1;12(6):982–5. doi: 10.1111/j.1526-4637.2011.01136.x 21627763 10.1111/j.1526-4637.2011.01136.x

[15] Paulozzi LJ, Kilbourne EM, Desai HA. Prescription drug monitoring programs and death rates from drug overdose. Pain Med. 2011 5;12(5):747–54. doi: 10.1111/j.1526-4637.2011.01062.x 21332934 10.1111/j.1526-4637.2011.01062.x

[16] Reifler LM, Droz D, Bailey JE, Schnoll SH, Fant R, Dart RC, et al Do prescription monitoring programs impact state trends in opioid abuse/misuse? Pain Med. 2012 3;13(3):434–42. doi: 10.1111/j.1526-4637.2012.01327.x 22299725 10.1111/j.1526-4637.2012.01327.x

[17] Knopf A. White House proposes $1 billion in new funding to treat opioid use disorders. Alcoholism & Drug Abuse Weekly. 2016 2 8;28(6):1–2.

[18] Van Zee A. The promotion and marketing of oxycontin: commercial triumph, public health tragedy. Am J Public Health. 2009 2;99(2):221–7. doi: 10.2105/AJPH.2007.131714 18799767 10.2105/AJPH.2007.131714 PMC2622774

[19] Bero L. Industry Sponsorship and Research Outcome: A Cochrane Review. JAMA Intern Med. 2013 4 8;173(7):580–1. doi: 10.1001/jamainternmed.2013.4190 23440226 10.1001/jamainternmed.2013.4190

[20] Campsall P, Colizza K, Straus S, Stelfox HT. Financial Relationships between Organizations That Produce Clinical Practice Guidelines and the Biomedical Industry: A Cross-Sectional Study. PLoS Med. 2016 5;13(5):e1002029 doi: 10.1371/journal.pmed.1002029 27244653 10.1371/journal.pmed.1002029 PMC4887051

[21] Choudhry NK, Stelfox HT, Detsky AS. Relationships between authors of clinical practice guidelines and the pharmaceutical industry. JAMA. 2002 2 6;287(5):612–7. 11829700 10.1001/jama.287.5.612

[22] Cosgrove L, Bursztajn HJ, Krimsky S, Anaya M, Walker J. Conflicts of Interest and Disclosure in the American Psychiatric Association’s Clinical Practice Guidelines. Psychother Psychosom. 2009 4 28;78(4):228–32. doi: 10.1159/000214444 19401623 10.1159/000214444

[23] Licurse A, Barber E, Joffe S, Gross C. The impact of disclosing financial ties in research and clinical care: a systematic review. Arch Intern Med. 2010 4 26;170(8):675–82. doi: 10.1001/archinternmed.2010.39 20421551 10.1001/archinternmed.2010.39

[24] Li T, Vedula SS, Hadar N, Parkin C, Lau J, Dickersin K. Innovations in Data Collection, Management, and Archiving for Systematic Reviews. Ann Intern Med. 2015 2 17;162(4):287 doi: 10.7326/M14-1603 25686168 10.7326/M14-1603

[25] Higgins JPT, Green S. Cochrane Handbook for Systematic Reviews of Interventions. Wiley; 2008 672 p.

[26] Levi-Minzi MA, Surratt HL, O’Grady CL, Kurtz SP. Finding what works: Predicting health or social service linkage in drug using, African American, female sex workers in Miami, FL. Health Care Women Int. 2016 7;37(7):744–59. doi: 10.1080/07399332.2016.1158262 26933839 10.1080/07399332.2016.1158262 PMC4905792

[27] Moher D, Liberati A, Tetzlaff J, Altman DG, PRISMA Group. Preferred reporting items for systematic reviews and meta-analyses: the PRISMA statement. PLoS Med. 2009 7 21;6(7):e1000097 doi: 10.1371/journal.pmed.1000097 19621072 10.1371/journal.pmed.1000097 PMC2707599

[28] Lang TA, Altman DG. Basic statistical reporting for articles published in biomedical journals: the “Statistical Analyses and Methods in the Published Literature” or the SAMPL Guidelines”. Handbook, European Association of Science Editors. 2013;23–6. 10.1016/j.ijnurstu.2014.09.006 25441757

[29] Mattick RP, Breen C, Kimber J, Davoli M. Buprenorphine maintenance versus placebo or methadone maintenance for opioid dependence. Cochrane Database Syst Rev. 2014 2 6;(2):CD002207 doi: 10.1002/14651858.CD002207.pub4 24500948 10.1002/14651858.CD002207.pub4 PMC10617756

[30] Strings Attached: CADTH’s Database Search Filters | CADTH.ca [Internet]. [cited 2017 Jan 26]. https://www.cadth.ca/resources/finding-evidence/strings-attached-cadths-database-search-filters

[31] Institute of Medicine, Board on Health Care Services, Committee on Standards for Developing Trustworthy Clinical Practice Guidelines. Clinical Practice Guidelines We Can Trust. National Academies Press; 2011. 290 p.

[32] Shea BJ, Grimshaw JM, Wells GA, Boers M, Andersson N, Hamel C, et al Development of AMSTAR: a measurement tool to assess the methodological quality of systematic reviews. BMC Med Res Methodol. 2007 2 15;7:10 doi: 10.1186/1471-2288-7-10 17302989 10.1186/1471-2288-7-10 PMC1810543

[33] Shea BJ, Hamel C, Wells GA, Bouter LM, Kristjansson E, Grimshaw J, et al AMSTAR is a reliable and valid measurement tool to assess the methodological quality of systematic reviews. J Clin Epidemiol. 2009 10;62(10):1013–20. doi: 10.1016/j.jclinepi.2008.10.009 19230606 10.1016/j.jclinepi.2008.10.009

[34] Pieper D, Buechter RB, Li L, Prediger B, Eikermann M. Systematic review found AMSTAR, but not R(evised)-AMSTAR, to have good measurement properties. J Clin Epidemiol. 2015/5;68(5):574–83. doi: 10.1016/j.jclinepi.2014.12.009 25638457 10.1016/j.jclinepi.2014.12.009

[35] Burda BU, Holmer HK, Norris SL. Limitations of A Measurement Tool to Assess Systematic Reviews (AMSTAR) and suggestions for improvement. Syst Rev. 2016 4 12;5:58 doi: 10.1186/s13643-016-0237-1 27072548 10.1186/s13643-016-0237-1 PMC4830078

[36] Popovich I, Windsor B, Jordan V, Showell M, Shea B, Farquhar CM. Methodological quality of systematic reviews in subfertility: a comparison of two different approaches. PLoS One. 2012 12 28;7(12):e50403 doi: 10.1371/journal.pone.0050403 23300526 10.1371/journal.pone.0050403 PMC3532502

[37] Sterne JAC, Sutton AJ, Ioannidis JPA, Terrin N, Jones DR, Lau J, et al Recommendations for examining and interpreting funnel plot asymmetry in meta-analyses of randomised controlled trials. BMJ. 2011 7 22;343:d4002 doi: 10.1136/bmj.d4002 21784880 10.1136/bmj.d4002

[38] Sharif MO, Janjua-Sharif FN, Sharif FNJ, Ali H, Ahmed F. Systematic reviews explained: AMSTAR-how to tell the good from the bad and the ugly. Oral Health Dent Manag. 2013 3;12(1):9–16. 23474576

[39] Liberati A, Altman DG, Tetzlaff J, Mulrow C, Gøtzsche PC, Ioannidis JPA, et al The PRISMA statement for reporting systematic reviews and meta-analyses of studies that evaluate healthcare interventions: explanation and elaboration. BMJ. 2009 7 21;339:b2700 doi: 10.1136/bmj.b2700 19622552 10.1136/bmj.b2700 PMC2714672

[40] Moher D, Shamseer L, Clarke M, Ghersi D, Liberati A, Petticrew M, et al Preferred reporting items for systematic review and meta-analysis protocols (PRISMA-P) 2015 statement. Syst Rev. 2015 1 1;4:1 doi: 10.1186/2046-4053-4-1 25554246 10.1186/2046-4053-4-1 PMC4320440

[41] Bryce S, Sloan E, Lee S, Ponsford J, Rossell S. Cognitive remediation in schizophrenia: A methodological appraisal of systematic reviews and meta-analyses. J Psychiatr Res. 2016 4;75:91–106. doi: 10.1016/j.jpsychires.2016.01.004 26828372 10.1016/j.jpsychires.2016.01.004

[42] Magellan Healthcare Clinical Practice Guideline Task Force (2015). Introduction to Magellan’s Adopted Clinical Practice Guidelines For the Assessment and Treatment of Patients With Substance Use Disorders.

[43] Ministry of Health (2014). New Zealand Practice Guidelines for Opioid Substitution Treatment.

[44] National Institute for Health and Care Excellence. (2007). Methadone and buprenorphine for the management of opioid dependence.

[45] The Management of Substance Use Disorders Work Group. (2015). Va/DoD Clinical Practice Guidelines For The Management of Substance Use disorders.

[46] The College of Physicians & Surgeons of Ontario. (2011). Methadone Maintenance Treatment Program Standards and Clinical Guidelines.

[47] Handford, C. (2011). Buprenorphine/Naloxone for Opioid dependence: Clinical practice guideline. Retrieved from https://www.cpso.on.ca/uploadedFiles/policies/guidelines/office/buprenorphine_naloxone_gdlns2011.pdf

[48] California Society of Addiction Medicine (2008). Guideline for Physicians Working in California Opioid Treatment Programs. San Francisco.

[49] Lingford-Hughes A., Welch S., Peters L., & Nutt D. (2012). BAP updated guidelines: Evidence-based guidelines for the pharmacological management of substance abuse, harmful use, addiction and comorbidity: Recommendations from BAP. *Journal of Psychopharmacology*, 26(7), 899–952. doi: 10.1177/0269881112444324 22628390 10.1177/0269881112444324

[50] Waller, R. (2014). Medication Assisted Treatment Guideline for Opioid Use Disorder.

[51] Ford, C., Halliday, K., Lawson, E., Browne, E., Modern, N., Lowe, C., … Watson, R. (2011). Royal College of general practitioners guidance for the use of substitute prescribing in the treatment of opioid dependence in primary care substance misuse management in general practice (SMMGP) the alliance written by: Guidance for the use of substitute. Retrieved from http://www.smmgp.org.uk/download/guidance/guidance004.pdf

[52] Amato L, Minozzi S, Davoli M, Vecchi S. Psychosocial combined with agonist maintenance treatments versus agonist maintenance treatments alone for treatment of opioid dependence. Cochrane Database Syst Rev. 2011 10 5;(10):CD004147 doi: 10.1002/14651858.CD004147.pub4 21975742 10.1002/14651858.CD004147.pub4

[53] Gowing L, Ali R, White JM. Buprenorphine for the management of opioid withdrawal. Cochrane Database Syst Rev. 2009 7 8;(3):CD002025 doi: 10.1002/14651858.CD002025.pub4 19588330 10.1002/14651858.CD002025.pub4

[54] Mattick RP, Breen C, Kimber J, Davoli M. Methadone maintenance therapy versus no opioid replacement therapy for opioid dependence. Cochrane Database Syst Rev. 2009 7 8;(3):CD002209 doi: 10.1002/14651858.CD002209.pub2 19588333

[55] Minozzi S, Amato L, Vecchi S, Davoli M, Kirchmayer U, Verster A. Oral naltrexone maintenance treatment for opioid dependence. Cochrane Database Syst Rev. 2011 2 16;(2):CD001333 doi: 10.1002/14651858.CD001333.pub3 16437431 10.1002/14651858.CD001333.pub2

[56] American Society of Addiction Medicine (2015). The National Practice Guideline For the Use of Medications in the Treatment of Addiction involving Opioid Use. 10.1097/ADM.0000000000000166 PMC4605275 26406300

[57] Riaz H, Raza S, Khan MS, Riaz IB, Krasuski RA. Impact of Funding Source on Clinical Trial Results Including Cardiovascular Outcome Trials. Am J Cardiol. 2015 12 15;116(12):1944–7. doi: 10.1016/j.amjcard.2015.09.034 26611124 10.1016/j.amjcard.2015.09.034

[58] Lexchin J, Bero LA, Djulbegovic B, Clark O. Pharmaceutical industry sponsorship and research outcome and quality: systematic review. BMJ. 2003 5 31;326(7400):1167–70. doi: 10.1136/bmj.326.7400.1167 12775614 10.1136/bmj.326.7400.1167 PMC156458

[59] Atakpo P, Vassar M. Publication bias in dermatology systematic reviews and meta-analyses. J Dermatol Sci. 2016 5;82(2):69–74. doi: 10.1016/j.jdermsci.2016.02.005 26925817 10.1016/j.jdermsci.2016.02.005

[60] Detweiler BN, Kollmorgen LE, Umberham BA, Hedin RJ, Vassar BM. Risk of bias and methodological appraisal practices in systematic reviews published in anaesthetic journals: a meta-epidemiological study. Anaesthesia. 2016 8;71(8):955–68. doi: 10.1111/anae.13520 27396249 10.1111/anae.13520

[61] Onishi A, Furukawa TA. Publication bias is underreported in systematic reviews published in high-impact-factor journals: metaepidemiologic study. J Clin Epidemiol. 2014 12;67(12):1320–6. doi: 10.1016/j.jclinepi.2014.07.002 25194857 10.1016/j.jclinepi.2014.07.002

[62] Roest AM, de Jonge P, Williams CD, de Vries YA, Schoevers RA, Turner EH. Reporting Bias in Clinical Trials Investigating the Efficacy of Second-Generation Antidepressants in the Treatment of Anxiety Disorders: A Report of 2 Meta-analyses. JAMA Psychiatry. 2015 5;72(5):500–10. doi: 10.1001/jamapsychiatry.2015.15 25806940 10.1001/jamapsychiatry.2015.15

[63] Tzoulaki I, Siontis KC, Evangelou E, Ioannidis JPA. Bias in associations of emerging biomarkers with cardiovascular disease. JAMA Intern Med. 2013 4 22;173(8):664–71. doi: 10.1001/jamainternmed.2013.3018 23529078 10.1001/jamainternmed.2013.3018

[64] Shamseer L, Moher D, Clarke M, Ghersi D, Liberati A, Petticrew M, et al Preferred reporting items for systematic review and meta-analysis protocols (PRISMA-P) 2015: elaboration and explanation. BMJ. 2015 1 2;349:g7647 doi: 10.1136/bmj.g7647 25555855 10.1136/bmj.g7647

[65] PROSPERO [Internet]. [cited 2017 Jan 26]. https://www.crd.york.ac.uk/PROSPERO/

[66] Mulrow CD. Rationale for systematic reviews. BMJ. 1994 9 3;309(6954):597–9. 8086953 10.1136/bmj.309.6954.597 PMC2541393

[67] Van der Wees P, Qaseem A, Kaila M, Ollenschlaeger G, Rosenfeld R, Board of Trustees of the Guidelines International Network (G-I-N). Prospective systematic review registration: perspective from the Guidelines International Network (G-I-N). Syst Rev. 2012 2 9;1:3 doi: 10.1186/2046-4053-1-3 22587933 10.1186/2046-4053-1-3 PMC3348674

[68] Whitehouse S. Text-S. 524-114th Congress (2015–2016): Comprehensive Addiction and Recovery Act of 2016. In: https://www.congress.gov [Internet]. 2016. http://maestro.abanet.org/trk/click?ref=z11aidwdq5_0-2ec8ex33eaf5x07551976&

